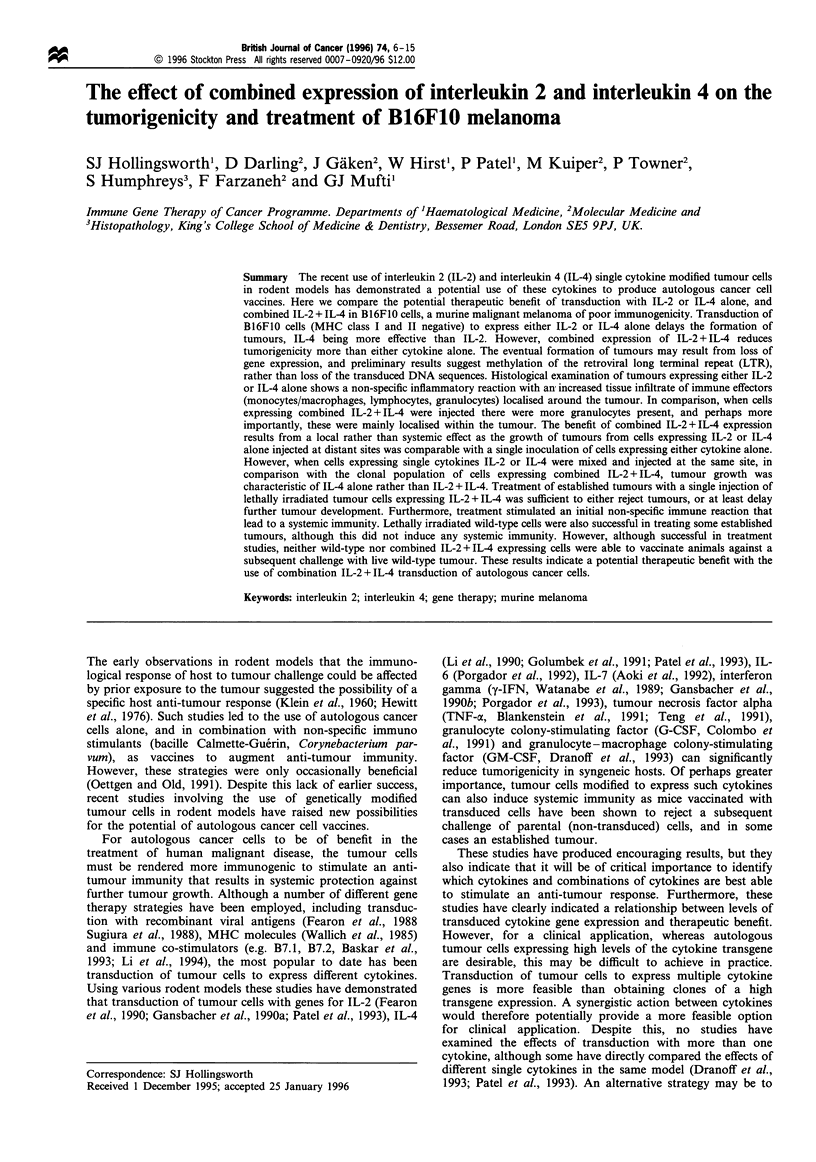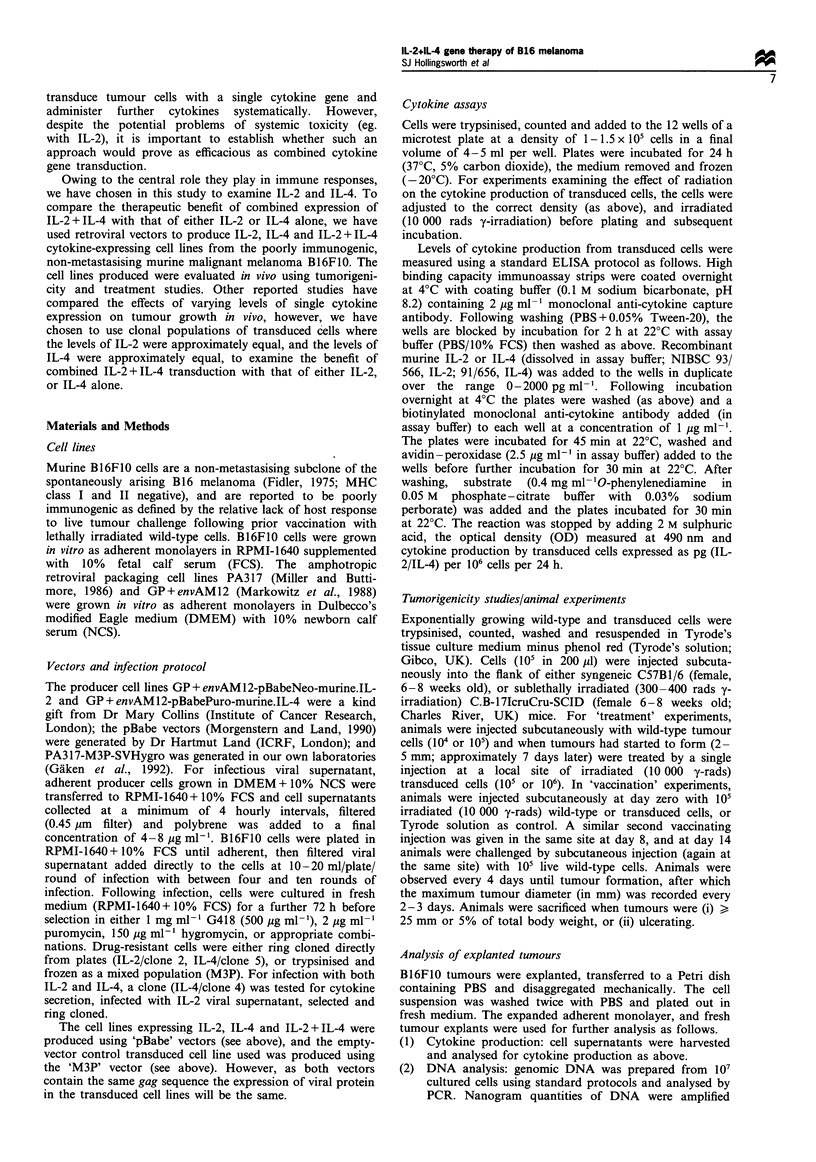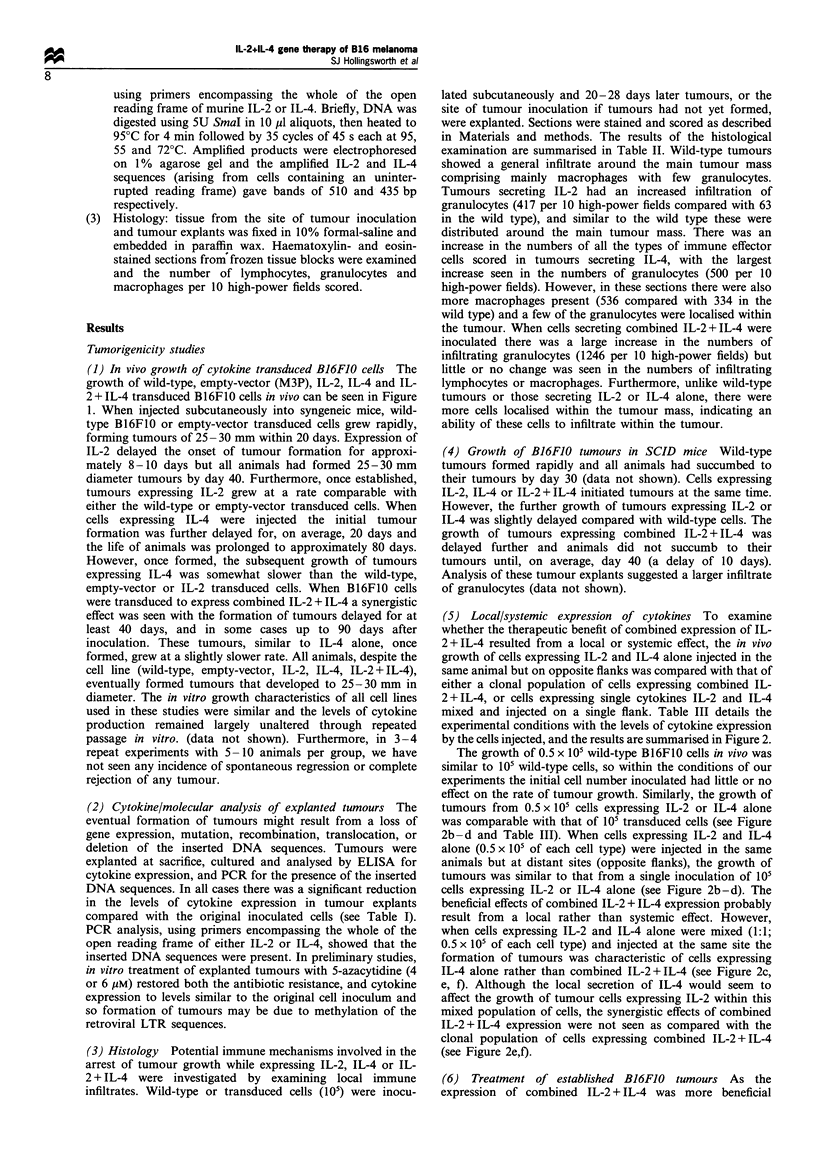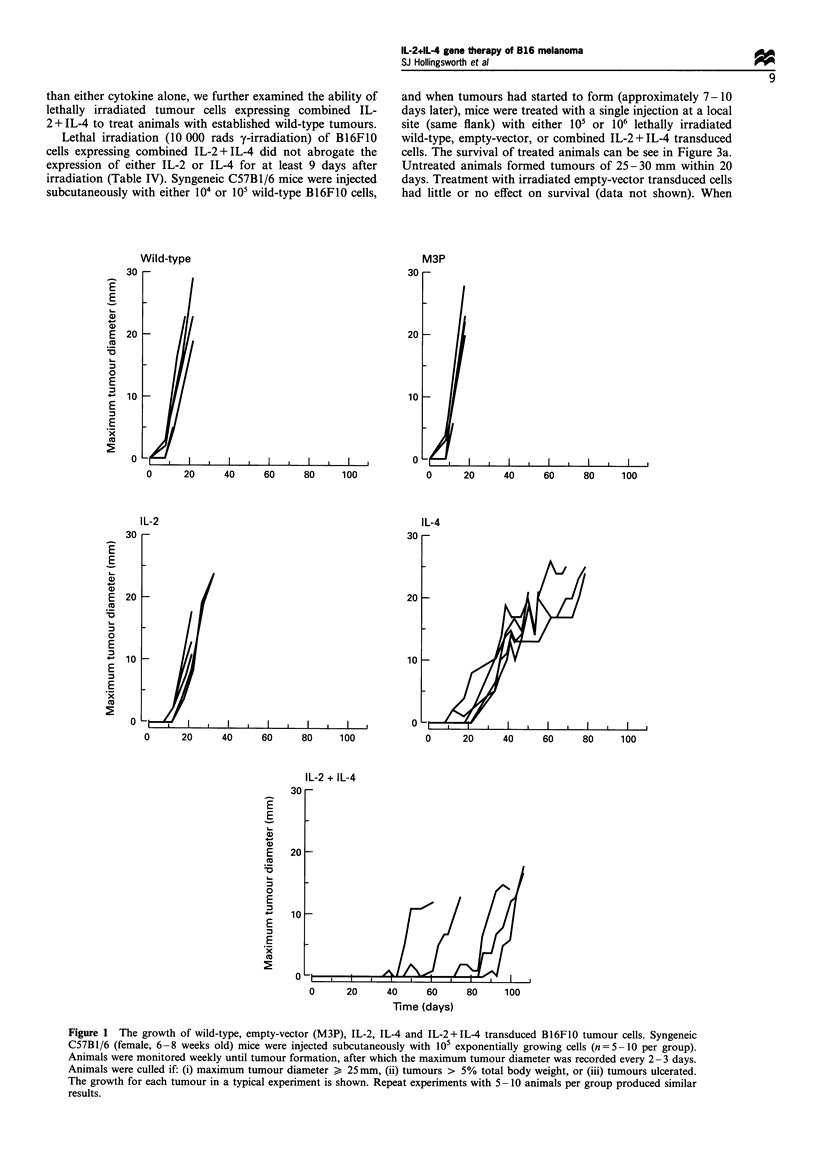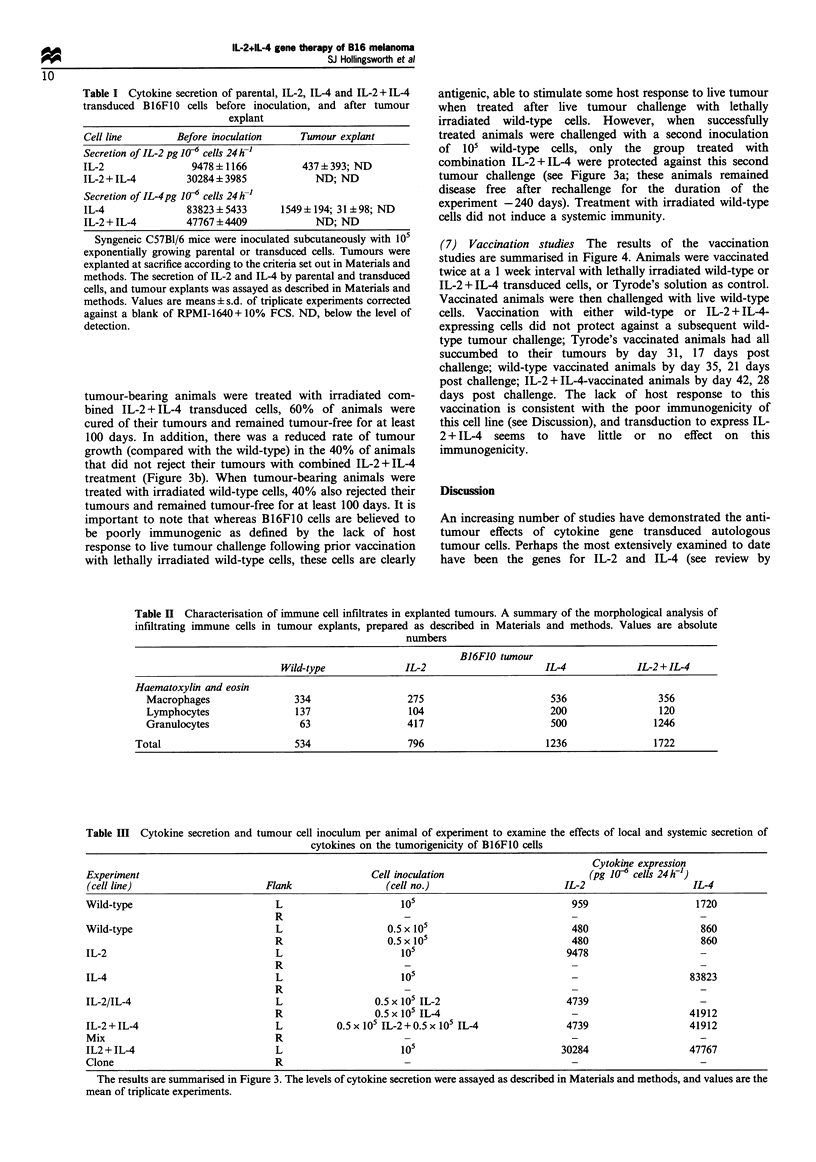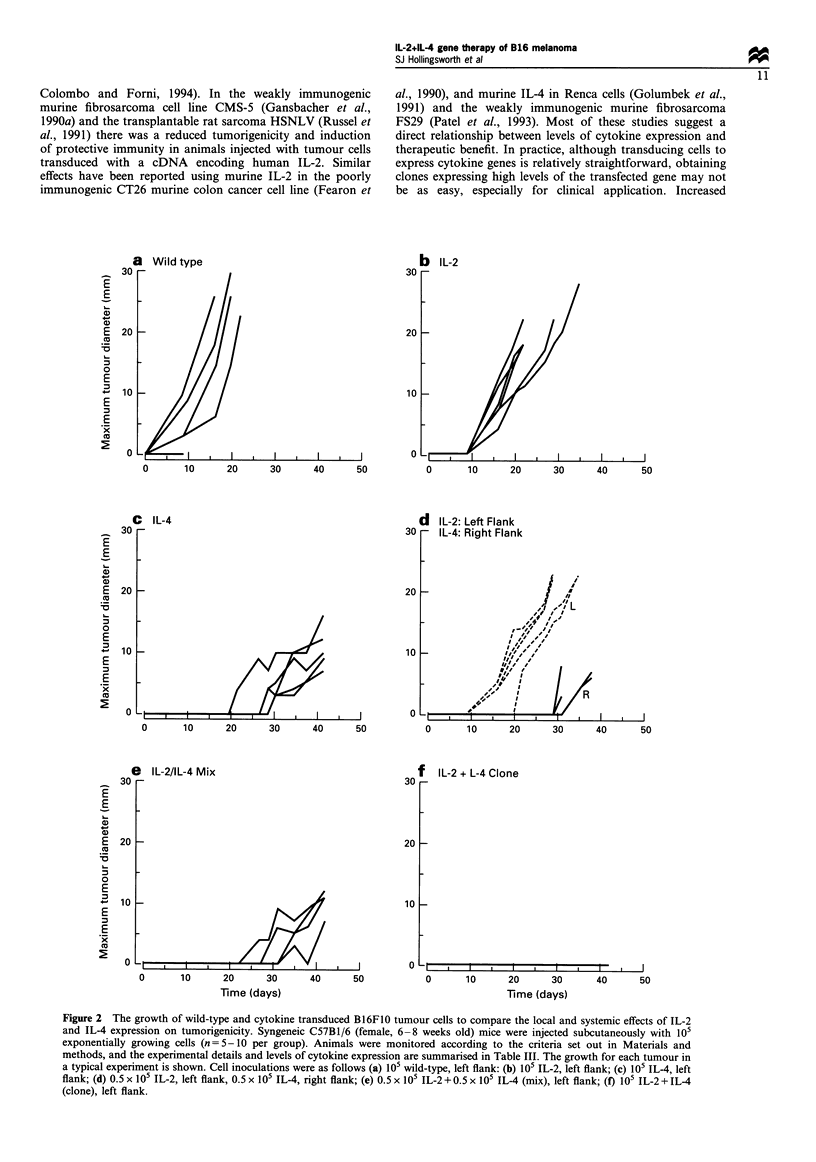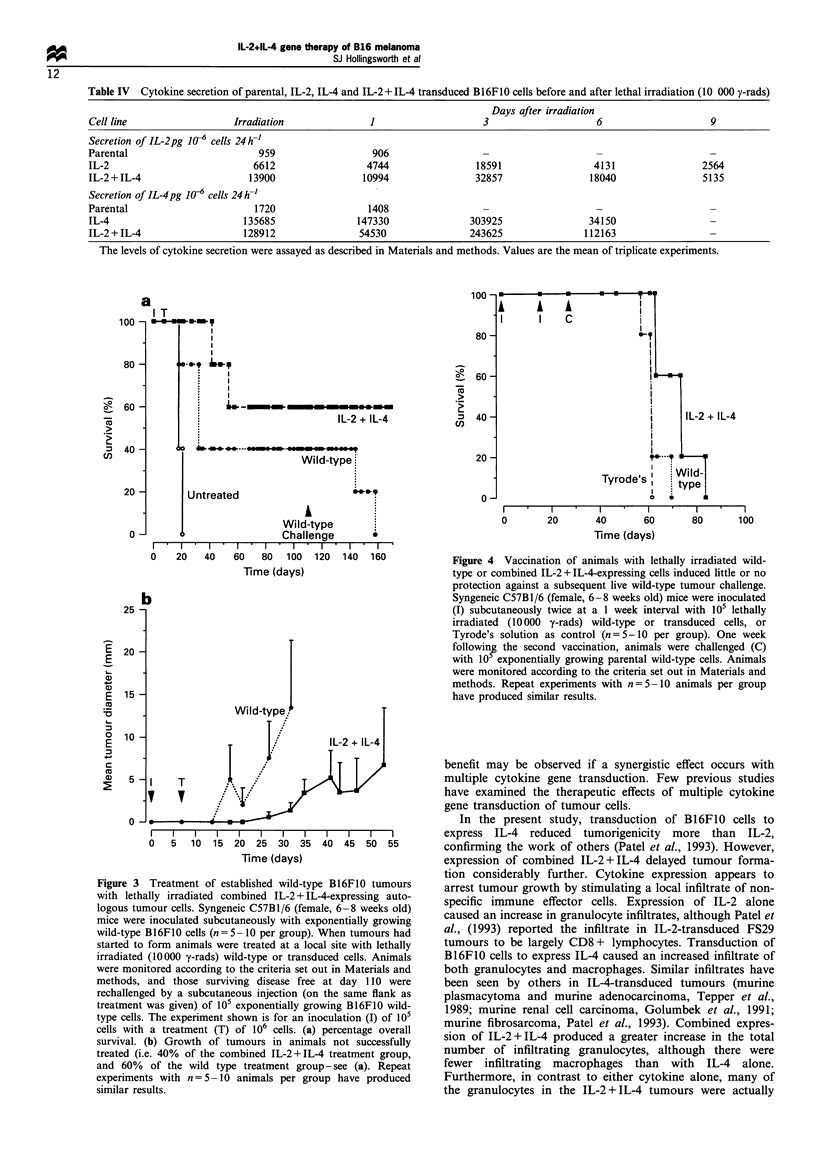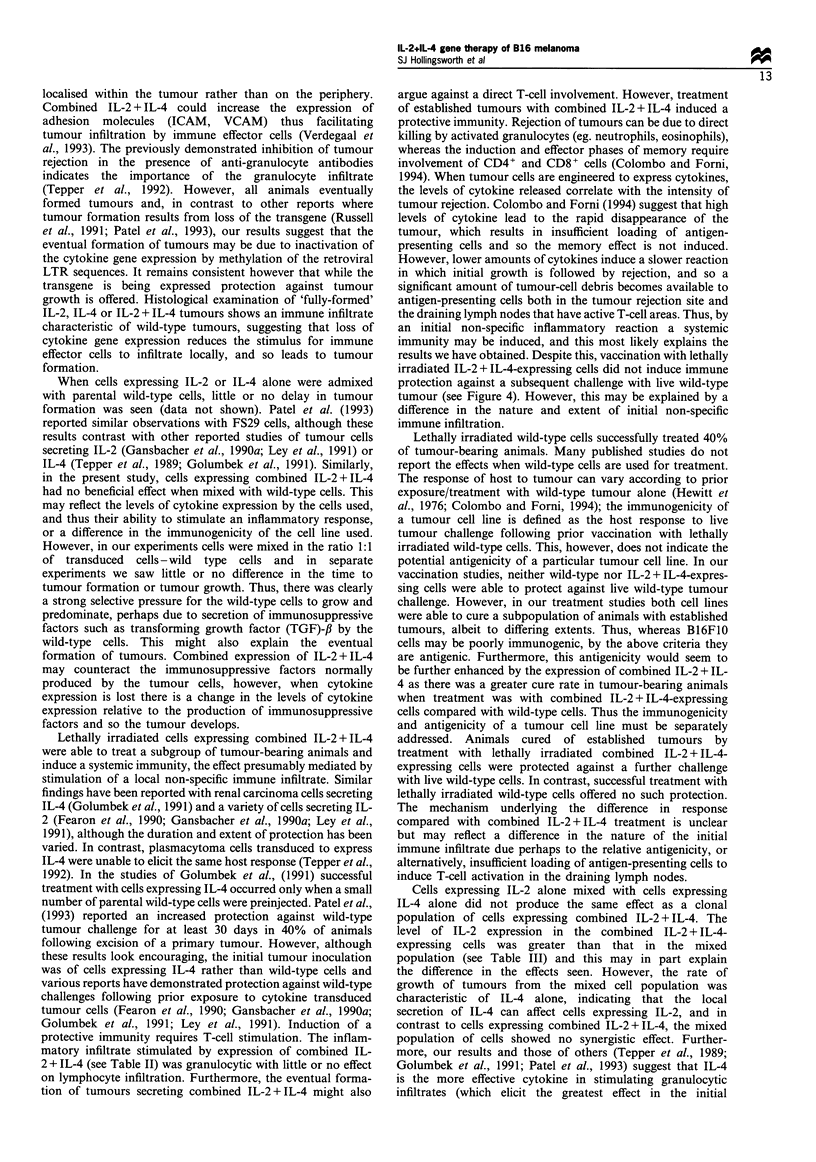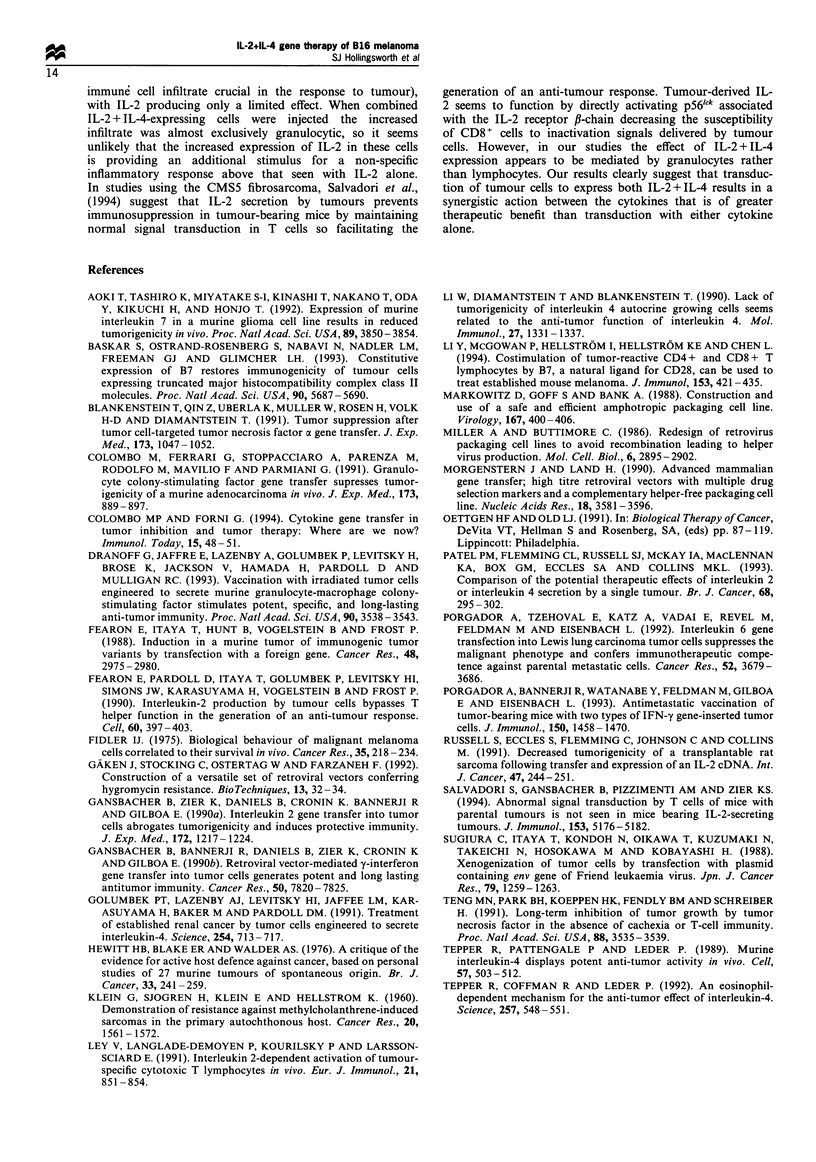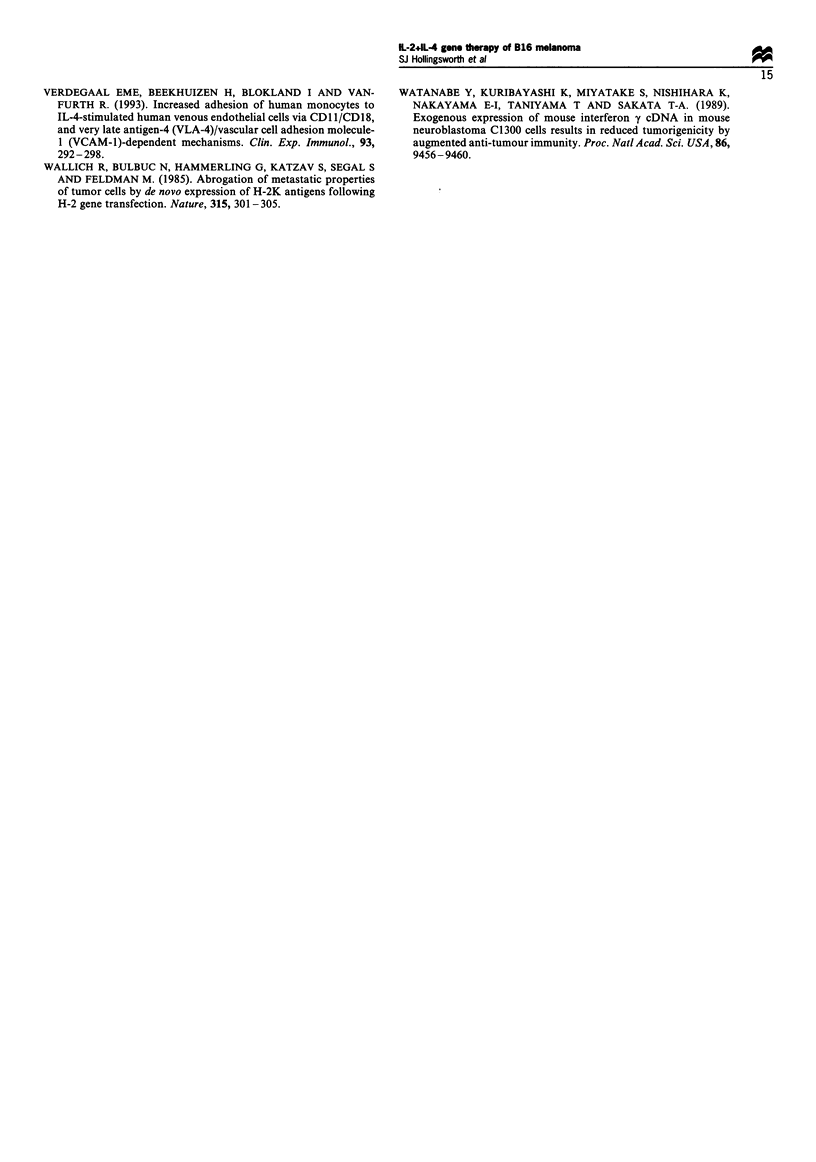# The effect of combined expression of interleukin 2 and interleukin 4 on the tumorigenicity and treatment of B16F10 melanoma.

**DOI:** 10.1038/bjc.1996.308

**Published:** 1996-07

**Authors:** S. J. Hollingsworth, D. Darling, J. Gäken, W. Hirst, P. Patel, M. Kuiper, P. Towner, S. Humphreys, F. Farzaneh, G. J. Mufti

**Affiliations:** Department of Haematological Medicine, King's College School of Medicine & Dentistry, London, UK.

## Abstract

The recent use of interleukin 2 (IL-2) and interleukin 4 (IL-4) single cytokine modified tumour cells in rodent models has demonstrated a potential use of these cytokines to produce autologous cancer cell vaccines. Here we compare the potential therapeutic benefit of transduction with IL-2 or IL-4 alone, and combined IL-2 + IL-4 in B16F10 cells, a murine malignant melanoma of poor immunogenicity. Transduction of B16F10 cells (MHC class I and II negative) to express either IL-2 or IL-4 alone delays the formation of tumours, IL-4 being more effective than IL-2. However, combined expression of IL-2 + IL-4 reduces tumorigenicity more than either cytokine alone. The eventual formation of tumours may result from loss of gene expression, and preliminary results suggest methylation of the retroviral long terminal repeat (LTR), rather than loss of the transduced DNA sequences. Histological examination of tumours expressing either IL-2 or IL-4 alone shows a non-specific inflammatory reaction with an increased tissue infiltrate of immune effectors (monocytes/macrophages, lymphocytes, granulocytes) localised around the tumour. In comparison, when cells expressing combined IL-2 + IL-4 were injected there were more granulocytes present, and perhaps more importantly, these were mainly localised within the tumour. The benefit of combined IL-2 + IL-4 expression results from a local rather than systemic effect as the growth of tumours from cells expressing IL-2 or IL-4 alone injected at distant sites was comparable with a single inoculation of cells expressing either cytokine alone. However, when cells expressing single cytokines IL-2 or IL-4 were mixed and injected at the same site, in comparison with the clonal population of cells expressing combined IL-2 + IL-4, tumour growth was characteristic of IL-4 alone rather than IL-2 + IL-4. Treatment of established tumours with a single injection of lethally irradiated tumour cells expressing IL-2 + IL-4 was sufficient to either reject tumours, or at least delay further tumour development. Furthermore, treatment stimulated an initial non-specific immune reaction that lead to a systemic immunity. Lethally irradiated wild-type cells were also successful in treating some established tumours, although this did not induce any systemic immunity. However, although successful in treatment studies, neither wild-type nor combined IL-2 + IL-4 expressing cells were able to vaccinate animals against a subsequent challenge with live wild-type tumour. These results indicate a potential therapeutic benefit with the use of combination IL-2 + IL-4 transduction of autologous cancer cells.


British Journal of Cancer (1996) 74, 6–15
© 1996 Stockton Press All rights reserved 0007–0920/96 $12.00

### The effect of combined expression of interleukin 2 and interleukin 4 on the tumorigenicity and treatment of B16F10 melanoma

SJ Hollingsworth[1], D Darling[2], J Gäken[2], W Hirst[1], P Patel[1], M Kuiper[2], P Towner[2], S Humphreys[3], F Farzaneh[2] and GJ Mufti[1]

*Immune Gene Therapy of Cancer Programme. Departments of [1]Haematological Medicine, [2]Molecular Medicine and [3]Histopathology, King's College School of Medicine & Dentistry, Bessemer Road, London SE5 9PJ, UK.*

**Summary** The recent use of interleukin 2 (IL-2) and interleukin 4 (IL-4) single cytokine modified tumour cells in rodent models has demonstrated a potential use of these cytokines to produce autologous cancer cell vaccines. Here we compare the potential therapeutic benefit of transduction with IL-2 or IL-4 alone, and combined IL-2 + IL-4 in B16F10 cells, a murine malignant melanoma of poor immunogenicity. Transduction of B16F10 cells (MHC class I and II negative) to express either IL-2 or IL-4 alone delays the formation of tumours, IL-4 being more effective than IL-2. However, combined expression of IL-2 + IL-4 reduces tumorigenicity more than either cytokine alone. The eventual formation of tumours may result from loss of gene expression, and preliminary results suggest methylation of the retroviral long terminal repeat (LTR), rather than loss of the transduced DNA sequences. Histological examination of tumours expressing either IL-2 or IL-4 alone shows a non-specific inflammatory reaction with an increased tissue infiltrate of immune effectors (monocytes/macrophages, lymphocytes, granulocytes) localised around the tumour. In comparison, when cells expressing combined IL-2 + IL-4 were injected there were more granulocytes present, and perhaps more importantly, these were mainly localised within the tumour. The benefit of combined IL-2 + IL-4 expression results from a local rather than systemic effect as the growth of tumours from cells expressing IL-2 or IL-4 alone injected at distant sites was comparable with a single inoculation of cells expressing either cytokine alone. However, when cells expressing single cytokines IL-2 or IL-4 were mixed and injected at the same site, in comparison with the clonal population of cells expressing combined IL-2 + IL-4, tumour growth was characteristic of IL-4 alone rather than IL-2 + IL-4. Treatment of established tumours with a single injection of lethally irradiated tumour cells expressing IL-2 + IL-4 was sufficient to either reject tumours, or at least delay further tumour development. Furthermore, treatment stimulated an initial non-specific immune reaction that lead to a systemic immunity. Lethally irradiated wild-type cells were also successful in treating some established tumours, although this did not induce any systemic immunity. However, although successful in treatment studies, neither wild-type nor combined IL-2 + IL-4 expressing cells were able to vaccinate animals against a subsequent challenge with live wild-type tumour. These results indicate a potential therapeutic benefit with the use of combination IL-2 + IL-4 transduction of autologous cancer cells.

**Keywords:** interleukin 2; interleukin 4; gene therapy; murine melanoma

The early observations in rodent models that the immunological response of host to tumour challenge could be affected by prior exposure to the tumour suggested the possibility of a specific host anti-tumour response (Klein *et al.*, 1960; Hewitt *et al.*, 1976). Such studies led to the use of autologous cancer cells alone, and in combination with non-specific immuno stimulants (bacille Calmette-Guérin, *Corynebacterium parvum*), as vaccines to augment anti-tumour immunity. However, these strategies were only occasionally beneficial (Oettgen and Old, 1991). Despite this lack of earlier success, recent studies involving the use of genetically modified tumour cells in rodent models have raised new possibilities for the potential of autologous cancer cell vaccines.

For autologous cancer cells to be of benefit in the treatment of human malignant disease, the tumour cells must be rendered more immunogenic to stimulate an anti-tumour immunity that results in systemic protection against further tumour growth. Although a number of different gene therapy strategies have been employed, including transduction with recombinant viral antigens (Fearon *et al.*, 1988 Sugiura *et al.*, 1988), MHC molecules (Wallich *et al.*, 1985) and immune co-stimulators (e.g. B7.1, B7.2, Baskar *et al.*, 1993; Li *et al.*, 1994), the most popular to date has been transduction of tumour cells to express different cytokines. Using various rodent models these studies have demonstrated that transduction of tumour cells with genes for IL-2 (Fearon *et al.*, 1990; Gansbacher *et al.*, 1990a; Patel *et al.*, 1993), IL-4 (Li *et al.*, 1990; Golumbek *et al.*, 1991; Patel *et al.*, 1993), IL-6 (Porgador *et al.*, 1992), IL-7 (Aoki *et al.*, 1992), interferon gamma (γ-IFN, Watanabe *et al.*, 1989; Gansbacher *et al.*, 1990*b*; Porgador *et al.*, 1993), tumour necrosis factor alpha (TNF-α, Blankenstein *et al.*, 1991; Teng *et al.*, 1991), granulocyte colony-stimulating factor (G-CSF, Colombo *et al.*, 1991) and granulocyte–macrophage colony-stimulating factor (GM-CSF, Dranoff *et al.*, 1993) can significantly reduce tumorigenicity in syngeneic hosts. Of perhaps greater importance, tumour cells modified to express such cytokines can also induce systemic immunity as mice vaccinated with transduced cells have been shown to reject a subsequent challenge of parental (non-transduced) cells, and in some cases an established tumour.

These studies have produced encouraging results, but they also indicate that it will be of critical importance to identify which cytokines and combinations of cytokines are best able to stimulate an anti-tumour response. Furthermore, these studies have clearly indicated a relationship between levels of transduced cytokine gene expression and therapeutic benefit. However, for a clinical application, whereas autologous tumour cells expressing high levels of the cytokine transgene are desirable, this may be difficult to achieve in practice. Transduction of tumour cells to express multiple cytokine genes is more feasible than obtaining clones of a high transgene expression. A synergistic action between cytokines would therefore potentially provide a more feasible option for clinical application. Despite this, no studies have examined the effects of transduction with more than one cytokine, although some have directly compared the effects of different single cytokines in the same model (Dranoff *et al.*, 1993; Patel *et al.*, 1993). An alternative strategy may be to

Correspondence: SJ Hollingsworth
Received 1 December 1995; accepted 25 January 1996

transduce tumour cells with a single cytokine gene and administer further cytokines systematically. However, despite the potential problems of systemic toxicity (eg. with IL-2), it is important to establish whether such an approach would prove as efficacious as combined cytokine gene transduction.

Owing to the central role they play in immune responses, we have chosen in this study to examine IL-2 and IL-4. To compare the therapeutic benefit of combined expression of IL-2 + IL-4 with that of either IL-2 or IL-4 alone, we have used retroviral vectors to produce IL-2, IL-4 and IL-2 + IL-4 cytokine-expressing cell lines from the poorly immunogenic, non-metastasising murine malignant melanoma B16F10. The cell lines produced were evaluated *in vivo* using tumorigenicity and treatment studies. Other reported studies have compared the effects of varying levels of single cytokine expression on tumour growth *in vivo*, however, we have chosen to use clonal populations of transduced cells where the levels of IL-2 were approximately equal, and the levels of IL-4 were approximately equal, to examine the benefit of combined IL-2 + IL-4 transduction with that of either IL-2, or IL-4 alone.

### Materials and Methods

### *Cell lines*

Murine B16F10 cells are a non-metastasising subclone of the spontaneously arising B16 melanoma (Fidler, 1975; MHC class I and II negative), and are reported to be poorly immunogenic as defined by the relative lack of host response to live tumour challenge following prior vaccination with lethally irradiated wild-type cells. B16F10 cells were grown *in vitro* as adherent monolayers in RPMI-1640 supplemented with 10% fetal calf serum (FCS). The amphotropic retroviral packaging cell lines PA317 (Miller and Buttimore, 1986) and GP + *env*AM12 (Markowitz *et al*., 1988) were grown *in vitro* as adherent monolayers in Dulbecco's modified Eagle medium (DMEM) with 10% newborn calf serum (NCS).

### *Vectors and infection protocol*

The producer cell lines GP + *env*AM12-pBabeNeo-murine.IL-2 and GP + *env*AM12-pBabePuro-murine.IL-4 were a kind gift from Dr Mary Collins (Institute of Cancer Research, London); the pBabe vectors (Morgenstern and Land, 1990) were generated by Dr Hartmut Land (ICRF, London); and PA317-M3P-SVHygro was generated in our own laboratories (Gäken *et al*., 1992). For infectious viral supernatant, adherent producer cells grown in DMEM + 10% NCS were transferred to RPMI-1640 + 10% FCS and cell supernatants collected at a minimum of 4 hourly intervals, filtered (0.45 $\mu$m filter) and polybrene was added to a final concentration of 4–8 $\mu$g ml$^{-1}$. B16F10 cells were plated in RPMI-1640 + 10% FCS until adherent, then filtered viral supernatant added directly to the cells at 10–20 ml/plate/round of infection with between four and ten rounds of infection. Following infection, cells were cultured in fresh medium (RPMI-1640 + 10% FCS) for a further 72 h before selection in either 1 mg ml$^{-1}$ G418 (500 $\mu$g ml$^{-1}$), 2 $\mu$g ml$^{-1}$ puromycin, 150 $\mu$g ml$^{-1}$ hygromycin, or appropriate combinations. Drug-resistant cells were either ring cloned directly from plates (IL-2/clone 2, IL-4/clone 5), or trypsinised and frozen as a mixed population (M3P). For infection with both IL-2 and IL-4, a clone (IL-4/clone 4) was tested for cytokine secretion, infected with IL-2 viral supernatant, selected and ring cloned.

The cell lines expressing IL-2, IL-4 and IL-2 + IL-4 were produced using 'pBabe' vectors (see above), and the empty-vector control transduced cell line used was produced using the 'M3P' vector (see above). However, as both vectors contain the same *gag* sequence the expression of viral protein in the transduced cell lines will be the same.

### *Cytokine assays*

Cells were trypsinised, counted and added to the 12 wells of a microtest plate at a density of $1-1.5 \times 10^5$ cells in a final volume of 4–5 ml per well. Plates were incubated for 24 h (37°C, 5% carbon dioxide), the medium removed and frozen (–20°C). For experiments examining the effect of radiation on the cytokine production of transduced cells, the cells were adjusted to the correct density (as above), and irradiated (10 000 rads $\gamma$-irradiation) before plating and subsequent incubation.

Levels of cytokine production from transduced cells were measured using a standard ELISA protocol as follows. High binding capacity immunoassay strips were coated overnight at 4°C with coating buffer (0.1 M sodium bicarbonate, pH 8.2) containing 2 $\mu$g ml$^{-1}$ monoclonal anti-cytokine capture antibody. Following washing (PBS + 0.05% Tween-20), the wells are blocked by incubation for 2 h at 22°C with assay buffer (PBS/10% FCS) then washed as above. Recombinant murine IL-2 or IL-4 (dissolved in assay buffer; NIBSC 93/566, IL-2; 91/656, IL-4) was added to the wells in duplicate over the range 0–2000 pg ml$^{-1}$. Following incubation overnight at 4°C the plates were washed (as above) and a biotinylated monoclonal anti-cytokine antibody added (in assay buffer) to each well at a concentration of 1 $\mu$g ml$^{-1}$. The plates were incubated for 45 min at 22°C, washed and avidin–peroxidase (2.5 $\mu$g ml$^{-1}$ in assay buffer) added to the wells before further incubation for 30 min at 22°C. After washing, substrate (0.4 mg ml$^{-1}$*O*-phenylenediamine in 0.05 M phosphate–citrate buffer with 0.03% sodium perborate) was added and the plates incubated for 30 min at 22°C. The reaction was stopped by adding 2 M sulphuric acid, the optical density (OD) measured at 490 nm and cytokine production by transduced cells expressed as pg (IL-2/IL-4) per $10^6$ cells per 24 h.

### *Tumorigenicity studies/animal experiments*

Exponentially growing wild-type and transduced cells were trypsinised, counted, washed and resuspended in Tyrode's tissue culture medium minus phenol red (Tyrode's solution; Gibco, UK). Cells ($10^5$ in 200 $\mu$l) were injected subcutaneously into the flank of either syngeneic C57B1/6 (female, 6–8 weeks old), or sublethally irradiated (300–400 rads $\gamma$-irradiation) C.B-17IcruCru-SCID (female 6–8 weeks old; Charles River, UK) mice. For 'treatment' experiments, animals were injected subcutaneously with wild-type tumour cells ($10^4$ or $10^5$) and when tumours had started to form (2–5 mm; approximately 7 days later) were treated by a single injection at a local site of irradiated (10 000 $\gamma$-rads) transduced cells ($10^5$ or $10^6$). In 'vaccination' experiments, animals were injected subcutaneously at day zero with $10^5$ irradiated (10 000 $\gamma$-rads) wild-type or transduced cells, or Tyrode solution as control. A similar second vaccinating injection was given in the same site at day 8, and at day 14 animals were challenged by subcutaneous injection (again at the same site) with $10^5$ live wild-type cells. Animals were observed every 4 days until tumour formation, after which the maximum tumour diameter (in mm) was recorded every 2–3 days. Animals were sacrificed when tumours were (i) $\geqslant$ 25 mm or 5% of total body weight, or (ii) ulcerating.

### *Analysis of explanted tumours*

B16F10 tumours were explanted, transferred to a Petri dish containing PBS and disaggregated mechanically. The cell suspension was washed twice with PBS and plated out in fresh medium. The expanded adherent monolayer, and fresh tumour explants were used for further analysis as follows.

(1) Cytokine production: cell supernatants were harvested and analysed for cytokine production as above.
(2) DNA analysis: genomic DNA was prepared from $10^7$ cultured cells using standard protocols and analysed by PCR. Nanogram quantities of DNA were amplified using primers encompassing the whole of the open reading frame of murine IL-2 or IL-4. Briefly, DNA was digested using 5U *Sma*I in 10 $\mu$l aliquots, then heated to 95°C for 4 min followed by 35 cycles of 45 s each at 95, 55 and 72°C. Amplified products were electrophoresed on 1% agarose gel and the amplified IL-2 and IL-4 sequences (arising from cells containing an uninterrupted reading frame) gave bands of 510 and 435 bp respectively.

(3) Histology: tissue from the site of tumour inoculation and tumour explants was fixed in 10% formal-saline and embedded in paraffin wax. Haematoxylin- and eosin-stained sections from frozen tissue blocks were examined and the number of lymphocytes, granulocytes and macrophages per 10 high-power fields scored.

### Results

*Tumorigenicity studies*

*(1) In vivo growth of cytokine transduced B16F10 cells* The growth of wild-type, empty-vector (M3P), IL-2, IL-4 and IL-2 + IL-4 transduced B16F10 cells *in vivo* can be seen in Figure 1. When injected subcutaneously into syngeneic mice, wild-type B16F10 or empty-vector transduced cells grew rapidly, forming tumours of 25–30 mm within 20 days. Expression of IL-2 delayed the onset of tumour formation for approximately 8–10 days but all animals had formed 25–30 mm diameter tumours by day 40. Furthermore, once established, tumours expressing IL-2 grew at a rate comparable with either the wild-type or empty-vector transduced cells. When cells expressing IL-4 were injected the initial tumour formation was further delayed for, on average, 20 days and the life of animals was prolonged to approximately 80 days. However, once formed, the subsequent growth of tumours expressing IL-4 was somewhat slower than the wild-type, empty-vector or IL-2 transduced cells. When B16F10 cells were transduced to express combined IL-2 + IL-4 a synergistic effect was seen with the formation of tumours delayed for at least 40 days, and in some cases up to 90 days after inoculation. These tumours, similar to IL-4 alone, once formed, grew at a slightly slower rate. All animals, despite the cell line (wild-type, empty-vector, IL-2, IL-4, IL-2 + IL-4), eventually formed tumours that developed to 25–30 mm in diameter. The *in vitro* growth characteristics of all cell lines used in these studies were similar and the levels of cytokine production remained largely unaltered through repeated passage *in vitro*. (data not shown). Furthermore, in 3–4 repeat experiments with 5–10 animals per group, we have not seen any incidence of spontaneous regression or complete rejection of any tumour.

*(2) Cytokine/molecular analysis of explanted tumours* The eventual formation of tumours might result from a loss of gene expression, mutation, recombination, translocation, or deletion of the inserted DNA sequences. Tumours were explanted at sacrifice, cultured and analysed by ELISA for cytokine expression, and PCR for the presence of the inserted DNA sequences. In all cases there was a significant reduction in the levels of cytokine expression in tumour explants compared with the original inoculated cells (see Table I). PCR analysis, using primers encompassing the whole of the open reading frame of either IL-2 or IL-4, showed that the inserted DNA sequences were present. In preliminary studies, *in vitro* treatment of explanted tumours with 5-azacytidine (4 or 6 $\mu$M) restored both the antibiotic resistance, and cytokine expression to levels similar to the original cell inoculum and so formation of tumours may be due to methylation of the retroviral LTR sequences.

*(3) Histology* Potential immune mechanisms involved in the arrest of tumour growth while expressing IL-2, IL-4 or IL-2 + IL-4 were investigated by examining local immune infiltrates. Wild-type or transduced cells ($10^5$) were inoculated subcutaneously and 20–28 days later tumours, or the site of tumour inoculation if tumours had not yet formed, were explanted. Sections were stained and scored as described in Materials and methods. The results of the histological examination are summarised in Table II. Wild-type tumours showed a general infiltrate around the main tumour mass comprising mainly macrophages with few granulocytes. Tumours secreting IL-2 had an increased infiltration of granulocytes (417 per 10 high-power fields compared with 63 in the wild type), and similar to the wild type these were distributed around the main tumour mass. There was an increase in the numbers of all the types of immune effector cells scored in tumours secreting IL-4, with the largest increase seen in the numbers of granulocytes (500 per 10 high-power fields). However, in these sections there were also more macrophages present (536 compared with 334 in the wild type) and a few of the granulocytes were localised within the tumour. When cells secreting combined IL-2 + IL-4 were inoculated there was a large increase in the numbers of infiltrating granulocytes (1246 per 10 high-power fields) but little or no change was seen in the numbers of infiltrating lymphocytes or macrophages. Furthermore, unlike wild-type tumours or those secreting IL-2 or IL-4 alone, there were more cells localised within the tumour mass, indicating an ability of these cells to infiltrate within the tumour.

*(4) Growth of B16F10 tumours in SCID mice* Wild-type tumours formed rapidly and all animals had succumbed to their tumours by day 30 (data not shown). Cells expressing IL-2, IL-4 or IL-2 + IL-4 initiated tumours at the same time. However, the further growth of tumours expressing IL-2 or IL-4 was slightly delayed compared with wild-type cells. The growth of tumours expressing combined IL-2 + IL-4 was delayed further and animals did not succumb to their tumours until, on average, day 40 (a delay of 10 days). Analysis of these tumour explants suggested a larger infiltrate of granulocytes (data not shown).

*(5) Local/systemic expression of cytokines* To examine whether the therapeutic benefit of combined expression of IL-2 + IL-4 resulted from a local or systemic effect, the *in vivo* growth of cells expressing IL-2 and IL-4 alone injected in the same animal but on opposite flanks was compared with that of either a clonal population of cells expressing combined IL-2 + IL-4, or cells expressing single cytokines IL-2 and IL-4 mixed and injected on a single flank. Table III details the experimental conditions with the levels of cytokine expression by the cells injected, and the results are summarised in Figure 2.

The growth of $0.5 \times 10^5$ wild-type B16F10 cells *in vivo* was similar to $10^5$ wild-type cells, so within the conditions of our experiments the initial cell number inoculated had little or no effect on the rate of tumour growth. Similarly, the growth of tumours from $0.5 \times 10^5$ cells expressing IL-2 or IL-4 alone was comparable with that of $10^5$ transduced cells (see Figure 2b–d and Table III). When cells expressing IL-2 and IL-4 alone ($0.5 \times 10^5$ of each cell type) were injected in the same animals but at distant sites (opposite flanks), the growth of tumours was similar to that from a single inoculation of $10^5$ cells expressing IL-2 or IL-4 alone (see Figure 2b–d). The beneficial effects of combined IL-2 + IL-4 expression probably result from a local rather than systemic effect. However, when cells expressing IL-2 and IL-4 alone were mixed (1:1; $0.5 \times 10^5$ of each cell type) and injected at the same site the formation of tumours was characteristic of cells expressing IL-4 alone rather than combined IL-2 + IL-4 (see Figure 2c, e, f). Although the local secretion of IL-4 would seem to affect the growth of tumour cells expressing IL-2 within this mixed population of cells, the synergistic effects of combined IL-2 + IL-4 expression were not seen as compared with the clonal population of cells expressing combined IL-2 + IL-4 (see Figure 2e,f).

*(6) Treatment of established B16F10 tumours* As the expression of combined IL-2 + IL-4 was more beneficial than either cytokine alone, we further examined the ability of lethally irradiated tumour cells expressing combined IL-2 + IL-4 to treat animals with established wild-type tumours.

Lethal irradiation (10 000 rads γ-irradiation) of B16F10 cells expressing combined IL-2 + IL-4 did not abrogate the expression of either IL-2 or IL-4 for at least 9 days after irradiation (Table IV). Syngeneic C57Bl/6 mice were injected subcutaneously with either $10^4$ or $10^5$ wild-type B16F10 cells, and when tumours had started to form (approximately 7–10 days later), mice were treated with a single injection at a local site (same flank) with either $10^5$ or $10^6$ lethally irradiated wild-type, empty-vector, or combined IL-2 + IL-4 transduced cells. The survival of treated animals can be see in Figure 3a. Untreated animals formed tumours of 25–30 mm within 20 days. Treatment with irradiated empty-vector transduced cells had little or no effect on survival (data not shown). When

**Figure 1** The growth of wild-type, empty-vector (M3P), IL-2, IL-4 and IL-2 + IL-4 transduced B16F10 tumour cells. Syngeneic C57Bl/6 (female, 6–8 weeks old) mice were injected subcutaneously with $10^5$ exponentially growing cells ($n = 5-10$ per group). Animals were monitored weekly until tumour formation, after which the maximum tumour diameter was recorded every 2–3 days. Animals were culled if: (i) maximum tumour diameter ⩾ 25 mm, (ii) tumours > 5% total body weight, or (iii) tumours ulcerated. The growth for each tumour in a typical experiment is shown. Repeat experiments with 5–10 animals per group produced similar results.

**Table I** Cytokine secretion of parental, IL-2, IL-4 and IL-2 + IL-4 transduced B16F10 cells before inoculation, and after tumour explant

| *Cell line* | *Before inoculation* | *Tumour explant* |
|---|---|---|
| *Secretion of IL-2 pg $10^{-6}$ cells 24 $h^{-1}$* | | |
| IL-2 | 9478 ± 1166 | 437 ± 393; ND |
| IL-2 + IL-4 | 30284 ± 3985 | ND; ND |
| *Secretion of IL-4 pg $10^{-6}$ cells 24 $h^{-1}$* | | |
| IL-4 | 83823 ± 5433 | 1549 ± 194; 31 ± 98; ND |
| IL-2 + IL-4 | 47767 ± 4409 | ND; ND |

Syngeneic C57Bl/6 mice were inoculated subcutaneously with $10^5$ exponentially growing parental or transduced cells. Tumours were explanted at sacrifice according to the criteria set out in Materials and methods. The secretion of IL-2 and IL-4 by parental and transduced cells, and tumour explants was assayed as described in Materials and methods. Values are means ± s.d. of triplicate experiments corrected against a blank of RPMI-1640 + 10% FCS. ND, below the level of detection.

tumour-bearing animals were treated with irradiated combined IL-2 + IL-4 transduced cells, 60% of animals were cured of their tumours and remained tumour-free for at least 100 days. In addition, there was a reduced rate of tumour growth (compared with the wild-type) in the 40% of animals that did not reject their tumours with combined IL-2 + IL-4 treatment (Figure 3b). When tumour-bearing animals were treated with irradiated wild-type cells, 40% also rejected their tumours and remained tumour-free for at least 100 days. It is important to note that whereas B16F10 cells are believed to be poorly immunogenic as defined by the lack of host response to live tumour challenge following prior vaccination with lethally irradiated wild-type cells, these cells are clearly antigenic, able to stimulate some host response to live tumour when treated after live tumour challenge with lethally irradiated wild-type cells. However, when successfully treated animals were challenged with a second inoculation of $10^5$ wild-type cells, only the group treated with combination IL-2 + IL-4 were protected against this second tumour challenge (see Figure 3a; these animals remained disease free after rechallenge for the duration of the experiment −240 days). Treatment with irradiated wild-type cells did not induce a systemic immunity.

*(7) Vaccination studies* The results of the vaccination studies are summarised in Figure 4. Animals were vaccinated twice at a 1 week interval with lethally irradiated wild-type or IL-2 + IL-4 transduced cells, or Tyrode's solution as control. Vaccinated animals were then challenged with live wild-type cells. Vaccination with either wild-type or IL-2 + IL-4-expressing cells did not protect against a subsequent wild-type tumour challenge; Tyrode's vaccinated animals had all succumbed to their tumours by day 31, 17 days post challenge; wild-type vaccinated animals by day 35, 21 days post challenge; IL-2 + IL-4-vaccinated animals by day 42, 28 days post challenge. The lack of host response to this vaccination is consistent with the poor immunogenicity of this cell line (see Discussion), and transduction to express IL-2 + IL-4 seems to have little or no effect on this immunogenicity.

### Discussion

An increasing number of studies have demonstrated the anti-tumour effects of cytokine gene transduced autologous tumour cells. Perhaps the most extensively examined to date have been the genes for IL-2 and IL-4 (see review by

**Table II** Characterisation of immune cell infiltrates in explanted tumours. A summary of the morphological analysis of infiltrating immune cells in tumour explants, prepared as described in Materials and methods. Values are absolute numbers

| | | *B16F10 tumour* | | |
|---|---|---|---|---|
| | *Wild-type* | *IL-2* | *IL-4* | *IL-2 + IL-4* |
| *Haematoxylin and eosin* | | | | |
| Macrophages | 334 | 275 | 536 | 356 |
| Lymphocytes | 137 | 104 | 200 | 120 |
| Granulocytes | 63 | 417 | 500 | 1246 |
| Total | 534 | 796 | 1236 | 1722 |

**Table III** Cytokine secretion and tumour cell inoculum per animal of experiment to examine the effects of local and systemic secretion of cytokines on the tumorigenicity of B16F10 cells

| *Experiment (cell line)* | *Flank* | *Cell inoculation (cell no.)* | *Cytokine expression (pg $10^{-6}$ cells 24 $h^{-1}$)* IL-2 | IL-4 |
|---|---|---|---|---|
| Wild-type | L | $10^5$ | 959 | 1720 |
| | R | – | – | – |
| Wild-type | L | $0.5 \times 10^5$ | 480 | 860 |
| | R | $0.5 \times 10^5$ | 480 | 860 |
| IL-2 | L | $10^5$ | 9478 | – |
| | R | – | – | – |
| IL-4 | L | $10^5$ | – | 83823 |
| | R | – | – | – |
| IL-2/IL-4 | L | $0.5 \times 10^5$ IL-2 | 4739 | – |
| | R | $0.5 \times 10^5$ IL-4 | – | 41912 |
| IL-2 + IL-4 | L | $0.5 \times 10^5$ IL-2 + $0.5 \times 10^5$ IL-4 | 4739 | 41912 |
| Mix | R | – | – | – |
| IL2 + IL-4 | L | $10^5$ | 30284 | 47767 |
| Clone | R | – | – | – |

The results are summarised in Figure 3. The levels of cytokine secretion were assayed as described in Materials and methods, and values are the mean of triplicate experiments.

Colombo and Forni, 1994). In the weakly immunogenic murine fibrosarcoma cell line CMS-5 (Gansbacher *et al.*, 1990*a*) and the transplantable rat sarcoma HSNLV (Russel *et al.*, 1991) there was a reduced tumorigenicity and induction of protective immunity in animals injected with tumour cells transduced with a cDNA encoding human IL-2. Similar effects have been reported using murine IL-2 in the poorly immunogenic CT26 murine colon cancer cell line (Fearon *et al.*, 1990), and murine IL-4 in Renca cells (Golumbek *et al.*, 1991) and the weakly immunogenic murine fibrosarcoma FS29 (Patel *et al.*, 1993). Most of these studies suggest a direct relationship between levels of cytokine expression and therapeutic benefit. In practice, although transducing cells to express cytokine genes is relatively straightforward, obtaining clones expressing high levels of the transfected gene may not be as easy, especially for clinical application. Increased

**Figure 2** The growth of wild-type and cytokine transduced B16F10 tumour cells to compare the local and systemic effects of IL-2 and IL-4 expression on tumorigenicity. Syngeneic C57Bl/6 (female, 6–8 weeks old) mice were injected subcutaneously with $10^5$ exponentially growing cells ($n=5-10$ per group). Animals were monitored according to the criteria set out in Materials and methods, and the experimental details and levels of cytokine expression are summarised in Table III. The growth for each tumour in a typical experiment is shown. Cell inoculations were as follows (**a**) $10^5$ wild-type, left flank: (**b**) $10^5$ IL-2, left flank; (**c**) $10^5$ IL-4, left flank; (**d**) $0.5\times10^5$ IL-2, left flank, $0.5\times10^5$ IL-4, right flank; (**e**) $0.5\times10^5$ IL-2 + $0.5\times10^5$ IL-4 (mix), left flank; (**f**) $10^5$ IL-2 + IL-4 (clone), left flank.

Table IV Cytokine secretion of parental, IL-2, IL-4 and IL-2 + IL-4 transduced B16F10 cells before and after lethal irradiation (10 000 γ-rads)

| Cell line | Irradiation | Days after irradiation 1 | 3 | 6 | 9 |
|---|---|---|---|---|---|
| *Secretion of IL-2 pg $10^{-6}$ cells 24 $h^{-1}$* | | | | | |
| Parental | 959 | 906 | – | – | – |
| IL-2 | 6612 | 4744 | 18591 | 4131 | 2564 |
| IL-2 + IL-4 | 13900 | 10994 | 32857 | 18040 | 5135 |
| *Secretion of IL-4 pg $10^{-6}$ cells 24 $h^{-1}$* | | | | | |
| Parental | 1720 | 1408 | – | – | – |
| IL-4 | 135685 | 147330 | 303925 | 34150 | – |
| IL-2 + IL-4 | 128912 | 54530 | 243625 | 112163 | – |

The levels of cytokine secretion were assayed as described in Materials and methods. Values are the mean of triplicate experiments.

Figure 3 Treatment of established wild-type B16F10 tumours with lethally irradiated combined IL-2 + IL-4-expressing autologous tumour cells. Syngeneic C57B1/6 (female, 6–8 weeks old) mice were inoculated subcutaneously with exponentially growing wild-type B16F10 cells ($n$ = 5–10 per group). When tumours had started to form animals were treated at a local site with lethally irradiated (10 000 γ-rads) wild-type or transduced cells. Animals were monitored according to the criteria set out in Materials and methods, and those surviving disease free at day 110 were rechallenged by a subcutaneous injection (on the same flank as treatment was given) of $10^5$ exponentially growing B16F10 wild-type cells. The experiment shown is for an inoculation (I) of $10^5$ cells with a treatment (T) of $10^6$ cells. (**a**) percentage overall survival. (**b**) Growth of tumours in animals not successfully treated (i.e. 40% of the combined IL-2 + IL-4 treatment group, and 60% of the wild type treatment group – see (**a**). Repeat experiments with $n$ = 5–10 animals per group have produced similar results.

Figure 4 Vaccination of animals with lethally irradiated wild-type or combined IL-2 + IL-4-expressing cells induced little or no protection against a subsequent live wild-type tumour challenge. Syngeneic C57B1/6 (female, 6–8 weeks old) mice were inoculated (I) subcutaneously twice at a 1 week interval with $10^5$ lethally irradiated (10 000 γ-rads) wild-type or transduced cells, or Tyrode's solution as control ($n$ = 5–10 per group). One week following the second vaccination, animals were challenged (C) with $10^5$ exponentially growing parental wild-type cells. Animals were monitored according to the criteria set out in Materials and methods. Repeat experiments with $n$ = 5–10 animals per group have produced similar results.

benefit may be observed if a synergistic effect occurs with multiple cytokine gene transduction. Few previous studies have examined the therapeutic effects of multiple cytokine gene transduction of tumour cells.

In the present study, transduction of B16F10 cells to express IL-4 reduced tumorigenicity more than IL-2, confirming the work of others (Patel *et al.*, 1993). However, expression of combined IL-2 + IL-4 delayed tumour formation considerably further. Cytokine expression appears to arrest tumour growth by stimulating a local infiltrate of non-specific immune effector cells. Expression of IL-2 alone caused an increase in granulocyte infiltrates, although Patel *et al.*, (1993) reported the infiltrate in IL-2-transduced FS29 tumours to be largely CD8+ lymphocytes. Transduction of B16F10 cells to express IL-4 caused an increased infiltrate of both granulocytes and macrophages. Similar infiltrates have been seen by others in IL-4-transduced tumours (murine plasmacytoma and murine adenocarcinoma, Tepper *et al.*, 1989; murine renal cell carcinoma, Golumbek *et al.*, 1991; murine fibrosarcoma, Patel *et al.*, 1993). Combined expression of IL-2 + IL-4 produced a greater increase in the total number of infiltrating granulocytes, although there were fewer infiltrating macrophages than with IL-4 alone. Furthermore, in contrast to either cytokine alone, many of the granulocytes in the IL-2 + IL-4 tumours were actually localised within the tumour rather than on the periphery. Combined IL-2 + IL-4 could increase the expression of adhesion molecules (ICAM, VCAM) thus facilitating tumour infiltration by immune effector cells (Verdegaal *et al.*, 1993). The previously demonstrated inhibition of tumour rejection in the presence of anti-granulocyte antibodies indicates the importance of the granulocyte infiltrate (Tepper *et al.*, 1992). However, all animals eventually formed tumours and, in contrast to other reports where tumour formation results from loss of the transgene (Russell *et al.*, 1991; Patel *et al.*, 1993), our results suggest that the eventual formation of tumours may be due to inactivation of the cytokine gene expression by methylation of the retroviral LTR sequences. It remains consistent however that while the transgene is being expressed protection against tumour growth is offered. Histological examination of 'fully-formed' IL-2, IL-4 or IL-2 + IL-4 tumours shows an immune infiltrate characteristic of wild-type tumours, suggesting that loss of cytokine gene expression reduces the stimulus for immune effector cells to infiltrate locally, and so leads to tumour formation.

When cells expressing IL-2 or IL-4 alone were admixed with parental wild-type cells, little or no delay in tumour formation was seen (data not shown). Patel *et al.* (1993) reported similar observations with FS29 cells, although these results contrast with other reported studies of tumour cells secreting IL-2 (Gansbacher *et al.*, 1990*a*; Ley *et al.*, 1991) or IL-4 (Tepper *et al.*, 1989; Golumbek *et al.*, 1991). Similarly, in the present study, cells expressing combined IL-2 + IL-4 had no beneficial effect when mixed with wild-type cells. This may reflect the levels of cytokine expression by the cells used, and thus their ability to stimulate an inflammatory response, or a difference in the immunogenicity of the cell line used. However, in our experiments cells were mixed in the ratio 1:1 of transduced cells – wild type cells and in separate experiments we saw little or no difference in the time to tumour formation or tumour growth. Thus, there was clearly a strong selective pressure for the wild-type cells to grow and predominate, perhaps due to secretion of immunosuppressive factors such as transforming growth factor (TGF)-$\beta$ by the wild-type cells. This might also explain the eventual formation of tumours. Combined expression of IL-2 + IL-4 may counteract the immunosuppressive factors normally produced by the tumour cells, however, when cytokine expression is lost there is a change in the levels of cytokine expression relative to the production of immunosuppressive factors and so the tumour develops.

Lethally irradiated cells expressing combined IL-2 + IL-4 were able to treat a subgroup of tumour-bearing animals and induce a systemic immunity, the effect presumably mediated by stimulation of a local non-specific immune infiltrate. Similar findings have been reported with renal carcinoma cells secreting IL-4 (Golumbek *et al.*, 1991) and a variety of cells secreting IL-2 (Fearon *et al.*, 1990; Gansbacher *et al.*, 1990*a*; Ley *et al.*, 1991), although the duration and extent of protection has been varied. In contrast, plasmacytoma cells transduced to express IL-4 were unable to elicit the same host response (Tepper *et al.*, 1992). In the studies of Golumbek *et al.*, (1991) successful treatment with cells expressing IL-4 occurred only when a small number of parental wild-type cells were preinjected. Patel *et al.*, (1993) reported an increased protection against wild-type tumour challenge for at least 30 days in 40% of animals following excision of a primary tumour. However, although these results look encouraging, the initial tumour inoculation was of cells expressing IL-4 rather than wild-type cells and various reports have demonstrated protection against wild-type challenges following prior exposure to cytokine transduced tumour cells (Fearon *et al.*, 1990; Gansbacher *et al.*, 1990*a*; Golumbek *et al.*, 1991; Ley *et al.*, 1991). Induction of a protective immunity requires T-cell stimulation. The inflammatory infiltrate stimulated by expression of combined IL-2 + IL-4 (see Table II) was granulocytic with little or no effect on lymphocyte infiltration. Furthermore, the eventual formation of tumours secreting combined IL-2 + IL-4 might also argue against a direct T-cell involvement. However, treatment of established tumours with combined IL-2 + IL-4 induced a protective immunity. Rejection of tumours can be due to direct killing by activated granulocytes (eg. neutrophils, eosinophils), whereas the induction and effector phases of memory require involvement of $CD4^+$ and $CD8^+$ cells (Colombo and Forni, 1994). When tumour cells are engineered to express cytokines, the levels of cytokine released correlate with the intensity of tumour rejection. Colombo and Forni (1994) suggest that high levels of cytokine lead to the rapid disappearance of the tumour, which results in insufficient loading of antigen-presenting cells and so the memory effect is not induced. However, lower amounts of cytokines induce a slower reaction in which initial growth is followed by rejection, and so a significant amount of tumour-cell debris becomes available to antigen-presenting cells both in the tumour rejection site and the draining lymph nodes that have active T-cell areas. Thus, by an initial non-specific inflammatory reaction a systemic immunity may be induced, and this most likely explains the results we have obtained. Despite this, vaccination with lethally irradiated IL-2 + IL-4-expressing cells did not induce immune protection against a subsequent challenge with live wild-type tumour (see Figure 4). However, this may be explained by a difference in the nature and extent of initial non-specific immune infiltration.

Lethally irradiated wild-type cells successfully treated 40% of tumour-bearing animals. Many published studies do not report the effects when wild-type cells are used for treatment. The response of host to tumour can vary according to prior exposure/treatment with wild-type tumour alone (Hewitt *et al.*, 1976; Colombo and Forni, 1994); the immunogenicity of a tumour cell line is defined as the host response to live tumour challenge following prior vaccination with lethally irradiated wild-type cells. This, however, does not indicate the potential antigenicity of a particular tumour cell line. In our vaccination studies, neither wild-type nor IL-2 + IL-4-expressing cells were able to protect against live wild-type tumour challenge. However, in our treatment studies both cell lines were able to cure a subpopulation of animals with established tumours, albeit to differing extents. Thus, whereas B16F10 cells may be poorly immunogenic, by the above criteria they are antigenic. Furthermore, this antigenicity would seem to be further enhanced by the expression of combined IL-2 + IL-4 as there was a greater cure rate in tumour-bearing animals when treatment was with combined IL-2 + IL-4-expressing cells compared with wild-type cells. Thus the immunogenicity and antigenicity of a tumour cell line must be separately addressed. Animals cured of established tumours by treatment with lethally irradiated combined IL-2 + IL-4-expressing cells were protected against a further challenge with live wild-type cells. In contrast, successful treatment with lethally irradiated wild-type cells offered no such protection. The mechanism underlying the difference in response compared with combined IL-2 + IL-4 treatment is unclear but may reflect a difference in the nature of the initial immune infiltrate due perhaps to the relative antigenicity, or alternatively, insufficient loading of antigen-presenting cells to induce T-cell activation in the draining lymph nodes.

Cells expressing IL-2 alone mixed with cells expressing IL-4 alone did not produce the same effect as a clonal population of cells expressing combined IL-2 + IL-4. The level of IL-2 expression in the combined IL-2 + IL-4-expressing cells was greater than that in the mixed population (see Table III) and this may in part explain the difference in the effects seen. However, the rate of growth of tumours from the mixed cell population was characteristic of IL-4 alone, indicating that the local secretion of IL-4 can affect cells expressing IL-2, and in contrast to cells expressing combined IL-2 + IL-4, the mixed population of cells showed no synergistic effect. Furthermore, our results and those of others (Tepper *et al.*, 1989; Golumbek *et al.*, 1991; Patel *et al.*, 1993) suggest that IL-4 is the more effective cytokine in stimulating granulocytic infiltrates (which elicit the greatest effect in the initial immune cell infiltrate crucial in the response to tumour), with IL-2 producing only a limited effect. When combined IL-2 + IL-4-expressing cells were injected the increased infiltrate was almost exclusively granulocytic, so it seems unlikely that the increased expression of IL-2 in these cells is providing an additional stimulus for a non-specific inflammatory response above that seen with IL-2 alone. In studies using the CMS5 fibrosarcoma, Salvadori *et al.*, (1994) suggest that IL-2 secretion by tumours prevents immunosuppression in tumour-bearing mice by maintaining normal signal transduction in T cells so facilitating the generation of an anti-tumour response. Tumour-derived IL-2 seems to function by directly activating $p56^{lck}$ associated with the IL-2 receptor $\beta$-chain decreasing the susceptibility of $CD8^+$ cells to inactivation signals delivered by tumour cells. However, in our studies the effect of IL-2 + IL-4 expression appears to be mediated by granulocytes rather than lymphocytes. Our results clearly suggest that transduction of tumour cells to express both IL-2 + IL-4 results in a synergistic action between the cytokines that is of greater therapeutic benefit than transduction with either cytokine alone.

### References

AOKI T, TASHIRO K, MIYATAKE S-I, KINASHI T, NAKANO T, ODA Y, KIKUCHI H, AND HONJO T. (1992). Expression of murine interleukin 7 in a murine glioma cell line results in reduced tumorigenicity *in vivo*. *Proc. Natl Acad. Sci. USA*, **89**, 3850–3854.

BASKAR S, OSTRAND-ROSENBERG S, NABAVI N, NADLER LM, FREEMAN GJ AND GLIMCHER LH. (1993). Constitutive expression of B7 restores immunogenicity of tumour cells expressing truncated major histocompatibility complex class II molecules. *Proc. Natl Acad. Sci. USA*, **90**, 5687–5690.

BLANKENSTEIN T, QIN Z, UBERLA K, MULLER W, ROSEN H, VOLK H-D AND DIAMANTSTEIN T. (1991). Tumor suppression after tumor cell-targeted tumor necrosis factor α gene transfer. *J. Exp. Med.*, **173**, 1047–1052.

COLOMBO M, FERRARI G, STOPPACCIARO A, PARENZA M, RODOLFO M, MAVILIO F AND PARMIANI G. (1991). Granulocyte colony-stimulating factor gene transfer supresses tumorigenicity of a murine adenocarcinoma *in vivo*. *J. Exp. Med.*, **173**, 889–897.

COLOMBO MP AND FORNI G. (1994). Cytokine gene transfer in tumor inhibition and tumor therapy: Where are we now? *Immunol. Today*, **15**, 48–51.

DRANOFF G, JAFFRE E, LAZENBY A, GOLUMBEK P, LEVITSKY H, BROSE K, JACKSON V, HAMADA H, PARDOLL D AND MULLIGAN RC. (1993). Vaccination with irradiated tumor cells engineered to secrete murine granulocyte-macrophage colony-stimulating factor stimulates potent, specific, and long-lasting anti-tumor immunity. *Proc. Natl Acad. Sci. USA*, **90**, 3538–3543.

FEARON E, ITAYA T, HUNT B, VOGELSTEIN B AND FROST P. (1988). Induction in a murine tumor of immunogenic tumor variants by transfection with a foreign gene. *Cancer Res.*, **48**, 2975–2980.

FEARON E, PARDOLL D, ITAYA T, GOLUMBEK P, LEVITSKY HI, SIMONS JW, KARASUYAMA H, VOGELSTEIN B AND FROST P. (1990). Interleukin-2 production by tumour cells bypasses T helper function in the generation of an anti-tumour response. *Cell*, **60**, 397–403.

FIDLER IJ. (1975). Biological behaviour of malignant melanoma cells correlated to their survival *in vivo*. *Cancer Res.*, **35**, 218–234.

GÄKEN J, STOCKING C, OSTERTAG W AND FARZANEH F. (1992). Construction of a versatile set of retroviral vectors conferring hygromycin resistance. *BioTechniques*, **13**, 32–34.

GANSBACHER B, ZIER K, DANIELS B, CRONIN K. BANNERJI R AND GILBOA E. (1990*a*). Interleukin 2 gene transfer into tumor cells abrogates tumorigenicity and induces protective immunity. *J. Exp. Med.*, **172**, 1217–1224.

GANSBACHER B, BANNERJI R, DANIELS B, ZIER K, CRONIN K AND GILBOA E. (1990*b*). Retroviral vector-mediated γ-interferon gene transfer into tumor cells generates potent and long lasting antitumor immunity. *Cancer Res.*, **50**, 7820–7825.

GOLUMBEK PT, LAZENBY AJ, LEVITSKY HI, JAFFEE LM, KARASUYAMA H, BAKER M AND PARDOLL DM. (1991). Treatment of established renal cancer by tumor cells engineered to secrete interleukin-4. *Science*, **254**, 713–717.

HEWITT HB, BLAKE ER AND WALDER AS. (1976). A critique of the evidence for active host defence against cancer, based on personal studies of 27 murine tumours of spontaneous origin. *Br. J. Cancer*, **33**, 241–259.

KLEIN G, SJOGREN H, KLEIN E AND HELLSTROM K. (1960). Demonstration of resistance against methylcholanthrene-induced sarcomas in the primary autochthonous host. *Cancer Res.*, **20**, 1561–1572.

LEY V, LANGLADE-DEMOYEN P, KOURILSKY P AND LARSSON-SCIARD E. (1991). Interleukin 2-dependent activation of tumour-specific cytotoxic T lymphocytes *in vivo*. *Eur. J. Immunol.*, **21**, 851–854.

LI W, DIAMANTSTEIN T AND BLANKENSTEIN T. (1990). Lack of tumorigenicity of interleukin 4 autocrine growing cells seems related to the anti-tumor function of interleukin 4. *Mol. Immunol.*, **27**, 1331–1337.

LI Y, MCGOWAN P, HELLSTRÖM I, HELLSTRÖM KE AND CHEN L. (1994). Costimulation of tumor-reactive CD4+ and CD8+ T lymphocytes by B7, a natural ligand for CD28, can be used to treat established mouse melanoma. *J. Immunol*, **153**, 421–435.

MARKOWITZ D, GOFF S AND BANK A. (1988). Construction and use of a safe and efficient amphotropic packaging cell line. *Virology*, **167**, 400–406.

MILLER A AND BUTTIMORE C. (1986). Redesign of retrovirus packaging cell lines to avoid recombination leading to helper virus production. *Mol. Cell. Biol.*, **6**, 2895–2902.

MORGENSTERN J AND LAND H. (1990). Advanced mammalian gene transfer; high titre retroviral vectors with multiple drug selection markers and a complementary helper-free packaging cell line. *Nucleic Acids Res.*, **18**, 3581–3596.

OETTGEN HF AND OLD LJ. (1991). In: *Biological Therapy of Cancer*, DeVita VT, Hellman S and Rosenberg, SA, (eds) pp. 87–119. Lippincott: Philadelphia.

PATEL PM, FLEMMING CL, RUSSELL SJ, MCKAY IA, MACLENNAN KA, BOX GM, ECCLES SA AND COLLINS MKL. (1993). Comparison of the potential therapeutic effects of interleukin 2 or interleukin 4 secretion by a single tumour. *Br. J. Cancer*, **68**, 295–302.

PORGADOR A, TZEHOVAL E, KATZ A, VADAI E, REVEL M, FELDMAN M AND EISENBACH L. (1992). Interleukin 6 gene transfection into Lewis lung carcinoma tumor cells suppresses the malignant phenotype and confers immunotherapeutic competence against parental metastatic cells. *Cancer Res.*, **52**, 3679–3686.

PORGADOR A, BANNERJI R, WATANABE Y, FELDMAN M, GILBOA E AND EISENBACH L. (1993). Antimetastatic vaccination of tumor-bearing mice with two types of IFN-γ gene-inserted tumor cells. *J. Immunol.*, **150**, 1458–1470.

RUSSELL S, ECCLES S, FLEMMING C, JOHNSON C AND COLLINS M. (1991). Decreased tumorigenicity of a transplantable rat sarcoma following transfer and expression of an IL-2 cDNA. *Int. J. Cancer*, **47**, 244–251.

SALVADORI S, GANSBACHER B, PIZZIMENTI AM AND ZIER KS. (1994). Abnormal signal transduction by T cells of mice with parental tumours is not seen in mice bearing IL-2-secreting tumours. *J. Immunol.*, **153**, 5176–5182.

SUGIURA C, ITAYA T, KONDOH N, OIKAWA T, KUZUMAKI N, TAKEICHI N, HOSOKAWA M AND KOBAYASHI H. (1988). Xenogenization of tumor cells by transfection with plasmid containing *env* gene of Friend leukaemia virus. *Jpn. J. Cancer Res.*, **79**, 1259–1263.

TENG MN, PARK BH, KOEPPEN HK, FENDLY BM AND SCHREIBER H. (1991). Long-term inhibition of tumor growth by tumor necrosis factor in the absence of cachexia or T-cell immunity. *Proc. Natl Acad. Sci. USA*, **88**, 3535–3539.

TEPPER R, PATTENGALE P AND LEDER P. (1989). Murine interleukin-4 displays potent anti-tumor activity *in vivo*. *Cell*, **57**, 503–512.

TEPPER R, COFFMAN R AND LEDER P. (1992). An eosinophil-dependent mechanism for the anti-tumor effect of interleukin-4. *Science*, **257**, 548–551.

VERDEGAAL EME, BEEKHUIZEN H, BLOKLAND I AND VAN-FURTH R. (1993). Increased adhesion of human monocytes to IL-4-stimulated human venous endothelial cells via CD11/CD18, and very late antigen-4 (VLA-4)/vascular cell adhesion molecule-1 (VCAM-1)-dependent mechanisms. *Clin. Exp. Immunol.*, **93**, 292–298.

WALLICH R, BULBUC N, HAMMERLING G, KATZAV S, SEGAL S AND FELDMAN M. (1985). Abrogation of metastatic properties of tumor cells by *de novo* expression of H-2K antigens following H-2 gene transfection. *Nature*, **315**, 301–305.

WATANABE Y, KURIBAYASHI K, MIYATAKE S, NISHIHARA K, NAKAYAMA E-I, TANIYAMA T AND SAKATA T-A. (1989). Exogenous expression of mouse interferon $\gamma$ cDNA in mouse neuroblastoma C1300 cells results in reduced tumorigenicity by augmented anti-tumour immunity. *Proc. Natl Acad. Sci. USA*, **86**, 9456–9460.